# Crystal structures of the DE*x*H-box RNA helicase DHX9

**DOI:** 10.1107/S2059798323007611

**Published:** 2023-10-20

**Authors:** Young-Tae Lee, E. Allen Sickmier, Simina Grigoriu, Jennifer Castro, P. Ann Boriack-Sjodin

**Affiliations:** a Accent Therapeutics, 1050 Waltham Street, Lexington, MA 02421, USA; McGill University, Canada

**Keywords:** DHX9, RNA helicases, DE*x*H box, ADP

## Abstract

DHX9 is a DE*x*H-box RNA helicase with versatile functions in transcription, translation, RNA processing and regulation of DNA replication. DHX9 has recently emerged as a promising target for oncology, but to date no mammalian structures have been published. Here, crystal structures of human, dog and cat DHX9 bound to ADP are reported. The three mammalian DHX9 structures share identical structural folds. Additionally, the overall architecture and the individual domain structures of DHX9 are highly conserved with those of MLE, the *Drosophila* orthologue of DHX9 previously solved in complex with RNA and a transition-state analogue of ATP. Due to differences in the bound substrates and global domain orientations, the localized loop conformations and occupancy of dsRNA-binding domain 2 (dsRBD2) differ between the mammalian DHX9 and MLE structures. The combined effects of the structural changes considerably alter the RNA-binding channel, providing an opportunity to compare active and inactive states of the helicase. Finally, the mammalian DHX9 structures provide a potential tool for structure-based drug-design efforts.

## Abstract 2

The first crystal structures of the human, dog and cat DHX9 proteins were determined. The structures of mammalian DHX9 proteins share a similar structural fold with the previously reported structure of the *Drosophila melanogaster* DHX9 orthologue MLE. The human DHX9 structure provides a useful starting point for structure-guided drug design of DHX9 inhibitors.

## 1. Introduction

DHX9 [DE*x*H-box helicase 9, also known as RNA helicase A (RHA) or nuclear DNA helicase II (NDH II); EC 3.6.4.13] is a DE*x*H-box RNA helicase which exhibits unwinding activity for both DNA and RNA substrates (Zhang & Grosse, 1994 ▸; Lee & Hurwitz, 1992 ▸). DHX9 is capable of unwinding both double-stranded DNA and RNA as well as RNA/DNA hybrids (Lee & Hurwitz, 1992 ▸), although it has a higher predisposition for RNA-based substrates. In addition, DHX9 can unwind and/or resolve secondary helical structures such as R-loops, D-loops, triplex DNA, and DNA-based and RNA-based G-quadruplexes (Chakraborty & Grosse, 2011 ▸). DHX9 has been implicated in several biological processes, including regulation of transcription, translation, replication, RNA splicing and processing, and DNA repair (reviewed in Gulliver *et al.*, 2021 ▸; Lee & Pelletier, 2016 ▸).

Genomic instability is a hallmark of cancer (Hanahan & Weinberg, 2011 ▸; da Costa *et al.*, 2023 ▸), and proteins involved in DNA-damage pathways are potential targets for cancer research. DHX9 can interact with and regulate a large variety of proteins, some of which are key proteins involved in DNA-damage response (DDR) pathways such as BRCA1, Ku86, WRN and γH2AX (Chakraborty & Hiom, 2021 ▸). In addition, given the delicate balance of R-loop formation and resolution in maintaining efficient transcription and replication, the ability of DHX9 to unwind aberrant R-loops is important in helping to maintain genomic stability (Chakraborty *et al.*, 2018 ▸). DHX9 has been shown to be overexpressed in many cancer types, and DHX9 depletion has been shown to result in antiproliferative activity of multiple cancer cell lines (Gulliver *et al.*, 2021 ▸). It has also been shown that long-term suppression of DHX9 activity by inducible shRNA knockdown had no detrimental effects on normal adult mice at the organismal level (Lee *et al.*, 2016 ▸), indicating a higher dependency on DHX9 in select tumor cells. Together, these findings highlight the potential of DHX9 as a target for drug-discovery efforts in oncology.

DHX9 is a large, multidomain protein whose RecA domains (RecA1 and RecA2), which are required for helicase activity, are bounded by multiple auxiliary domains; these include two RNA-binding domains (dsRBD1 and dsRBD2) and a minimal transactivation domain (MTAD) N-terminal to the helicase core, and a helicase-associated domain (HA2), an oligonucleotide/oligosaccharide-binding fold (OB) and an RGG box C-terminal to the RecA domains. As reported for other DE*x*H helicases, DHX9 can hydrolyze any nucleotide triphosphates to unwind substrates (Lee & Hurwitz, 1992 ▸) and the nucleotide-binding motifs (motifs I–IV; Walker *et al.*, 1982 ▸) are highly conserved. The crystal structure of a truncated construct of MLE, the *Drosophila melanogaster* orthologue of DHX9, was solved in complex with single-stranded RNA and the transition-state mimic ADP–AlF_4_. This structure revealed that an N- and C-terminally truncated construct formed a well defined globular protein core consisting of multiple substructures and identified the structural elements that are responsible for RNA binding (Prabu *et al.*, 2015 ▸). More recently, additional structures of MLE have been determined by cryo-EM that further elucidate the mechanism of unwinding (Jagtap *et al.*, 2022 ▸). However, a structure of a mammalian DHX9 that includes all of the domains necessary for helicase activity has not yet been published. The crystal structure of the RecA1 domain of human DHX9 has previously been determined (Schütz *et al.*, 2010 ▸) and structures of the RNA-binding domains in apo and RNA-bound states have been solved by NMR (Nagata *et al.*, 2012 ▸) and crystallography (Fu & Yuan, 2013 ▸), respectively.

This work presents the first crystal structures of DHX9 from three mammalian species: human, dog and cat. All structures were solved in the presence of the nucleotide reaction product ADP. Overall, the structures reveal that the individual domain structures seen in *Drosophila* MLE are conserved in higher species. However, important differences are seen in the relative orientations of the auxiliary domain and helicase substructures and in select structural features of the helicase due to differences in the bound ligands or substrate. These differences also provide insights into the conformational changes that occur between active and inactive states of the enzyme. Given the interest in DHX9 as a therapeutic target, the structures presented here provide important information for future drug-discovery efforts.

## 2. Methods

### 2.1. Cloning, protein expression and purification

Human DHX9 (amino acids 150–1150; UniProt entry Q08211), dog DHX9 (amino acids 151–1151; UniProt entry A0A8C0M1F0) and cat DHX9 (amino acids 151–1151; UniProt entry A0A337SGK2) with a C-terminal FLAG tag were cloned into the pFastBac1 vector (Invitrogen). For protein expression, Sf9 cells (2–2.5 × 10^6^ cells ml^−1^) were infected with virus at a 1:100(*v*:*v*) ratio and cultured for 72 h at 27°C. The cells were harvested by centrifugation at 800*g* at 4°C and stored at −80°C until purification. For purification, the cells were resuspended in binding buffer (25 m*M* HEPES pH 7.5, 300 m*M* NaCl, 2 m*M* TCEP; 5 ml per gram of cells) supplemented with protease-inhibitor cocktail (Roche). The cells were lysed using a high-pressure homogenizer (ATS Engineering) at 20 MPa once and 40 MPa twice. The lysed cells were centrifuged at 13 600*g* for 30 min at 4°C. Centrifugation was repeated for the supernatant. The supernatant was loaded onto anti-FLAG affinity gel (Sigma) pre-equilibrated with binding buffer. The mixture was placed on a rotator for 2 h at 4°C. The resin was washed with binding buffer, followed by binding buffer with an additional 5 m*M* ATP and 10 m*M* MgCl_2_. The resin was then washed again with binding buffer and the target protein was eluted with 250 µg ml^−1^ FLAG peptide in binding buffer. The eluted protein was concentrated and loaded onto a Superdex 200 16/600 column (GE Healthcare) pre-equilibrated with binding buffer. The fractions containing the protein were pooled, concentrated to >20 mg ml^−1^, flash-frozen in liquid nitrogen and stored at −80°C until further use.

### 2.2. Melting-temperature measurements

The proteins were diluted in 25 m*M* HEPES pH 7.5, 300 m*M* NaCl, 2 m*M* TCEP to a final concentration of 1 mg ml^−1^. SYPRO Orange dye (Supelco) was diluted 50-fold in the same buffer and added to the proteins in a 1:15(*v*/*v*) ratio. Melting temperatures were measured on a LightCycler 480 II (Roche) in triplicate for each protein. Temperature was scanned from 25 to 95°C at a speed of 1°C min^−1^. The excitation and emission wavelengths were set to 490 and 575 nm, respectively. Melting temperatures were determined from the first-derivative curves.

### 2.3. Crystallization and data collection

Initial rod-shaped crystals of cat DHX9 were found in 100 m*M* Tris pH 8.0, 150 m*M* ammonium sulfate, 15%(*w*/*v*) PEG 4000, 5 m*M* ADP, 1.5 m*M* MgCl_2_. Similar rod-shaped crystals of dog DHX9 were found in 100 m*M* MES pH 6.0, 200 m*M* lithium sulfate, 20%(*w*/*v*) PEG 4000, 5 m*M* ADP, 1.5 m*M* MgCl_2_. Subsequent optimization using streak-seeding and fine screening around the MgCl_2_ concentration resulted in improved crystal reproducibility, morphology and diffraction. The cat and dog DHX9 crystals used for structure determination were obtained using streak-seeding in the original conditions with 50 m*M* MgCl_2_. Human DHX9 crystals were obtained by streak-seeding with optimized crystals of either cat or dog DHX9. The crystallization methods for human, dog and cat DHX9 are summarized in Table 1 ▸. For data collection, all crystals were cryoprotected using reservoir solution containing 15–20%(*v*/*v*) ethylene glycol and flash-cooled in liquid nitrogen.

### 2.4. Data processing, structure refinement and analysis

Integration of the diffraction data was conducted using *XDS* (Kabsch, 2010 ▸) for dog and cat DHX9 and *xia*2 -3dii (Winter, 2010 ▸) for human DHX9; scaling and merging were performed using *AIMLESS* (Evans, 2011 ▸). The structure of dog DHX9 was solved by molecular replacement in *Phaser *(McCoy *et al.*, 2007 ▸) using the crystal structure of MLE (PDB entry 5aor) as the search model. The cat and human DHX9 structures were solved by molecular replacement using the structure of dog DHX9 as a search model. Iterative cycles of refinement and manual model building were carried out using *REFMAC*5 (Murshudov *et al.*, 2011 ▸) and *Coot* (Emsley *et al.*, 2010 ▸), respectively. ADP, Mg^2+^ and waters were placed into the *F*_o_ − *F*_c_ density map using *Coot*. Structural models were validated using the wwPDB validation server (https://validate.wwpdb.org) and deposited in the Protein Data Bank (human DHX9, PDB entry 8szp; cat DHX9, PDB entry 8szq; dog DHX9, PDB entry 8szr). Data-collection and refinement statistics for the structures are summarized in Tables 2 ▸ and 3 ▸. Structural analysis was performed and figures were produced using *PyMOL* (Schrodinger). Calculations of r.m.s.d. and structural alignments were performed using the *align* command in *PyMOL* without and with rejecting outliers, respectively.

## 3. Results

### 3.1. Expression, purification and crystallization of human, dog and cat DHX9

**Figure 1:**
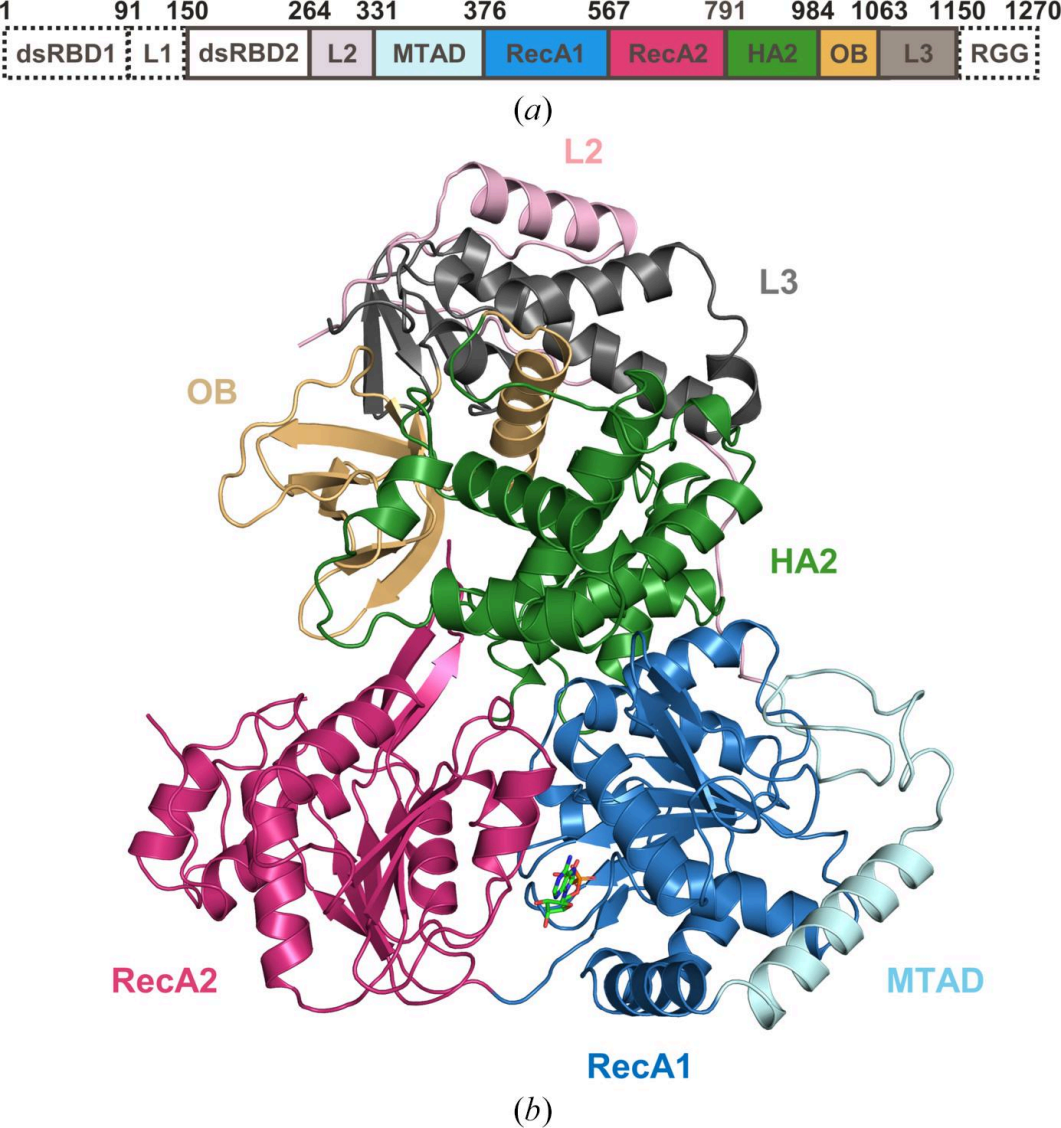
Crystal structure of human DHX9 bound to ADP. (*a*) Domain structure of human DHX9. Domains included in the crystallization construct (amino acids 150–1150) are outlined with solid lines. dsRBD1, dsRNA-binding domain 1; L1, linker 1; dsRBD2, dsRNA-binding domain 2; L2, linker 2; MTAD, minimal transactivation domain; RecA1, RecA-like domain 1; RecA2, RecA-like domain 2; HA2, helicase-associated 2 domain; OB, OB-fold domain; L3, linker 3; RGG, Arg-Gly-Gly-rich domain. (*b*) Structure of human DHX9 (ribbon) bound to ADP (green sticks). The color scheme for individual domains in Fig. 1 ▸(*a*) is applied to the ribbon representation in Fig. 1 ▸(*b*).

Expression and purification of full-length human DHX9 were attempted using both mammalian and insect cell expression systems, but protein of suitable purity and yield for crystallization efforts could not be obtained (data not shown). A truncated construct (Fig. 1 ▸ *a*), based on the successful MLE structure (PDB entry 5aor; Prabu *et al.*, 2015 ▸), was expressed in insect cells and purified to homogeneity (Supplementary Fig. S1*a*). Truncated human DHX9 (amino acids 150–1150) was catalytically active in both ATP-hydrolysis and RNA-unwinding assays (Gotur *et al.*, 2023 ▸), which is consistent with the activity results seen for the equivalent construct of MLE (Prabu *et al.*, 2015 ▸). Broad crystallization screening with human DHX9 alone and in the presence of nucleotide analogues (ADP, ATPγS and ADP–AlF_4_) using multiple commercially available crystallization screens did not produce protein crystals that could be optimized.

Because the initial efforts to crystallize human DHX9 were unsuccessful, mammalian orthologues were investigated. Multiple sequences were compared to identify species orthologues with high sequence identity to human DHX9. Dog and cat DHX9 were selected for protein production, as each was highly homologous to the human protein with sequence identities of 94.4% and 94.7% for the full-length dog and cat proteins, respectively (Supplementary Fig. S2). Truncated constructs for each species were made replicating the MLE and human DHX9 constructs and purified to homogeneity (Supplementary Figs. S1*b* and S1*c*). Interestingly, the cat and dog DHX9 constructs were found to have higher melting temperatures (dog DHX9, 58.7°C; cat DHX9, 60.6°C) than the human construct (57.4°C) (Supplementary Fig. S3) and the melting temperatures of all constructs increased by 1–2°C in the presence of nucleotide and magnesium (data not shown). Crystals were obtained in the presence of ADP and Mg^2+^ for both cat and dog DHX9. Streak-seeding greatly improved the crystallization reproducibility and the diffraction quality of both proteins. As a result, structures were solved at 2.97 and 2.71 Å resolution for dog and cat DHX9, respectively. Both proteins crystallized in space group *P*4_3_2_1_2 (one molecule per asymmetric unit) with extremely similar unit-cell constants, despite differences in pH, salt type and the amount of salt in the crystallization conditions (Tables 1 ▸ and 2 ▸).

As the structure of human DHX9 was of the greatest interest, seeds prepared from crystals of the species ortho­logues were utilized for crystallization efforts with the human protein. Using streak-seeding techniques, diffraction-quality crystals of human DHX9 were identified in the presence of ADP and Mg^2+^ in similar precipitant conditions as dog DHX9 and a 2.62 Å resolution structure of human DHX9 was determined. Despite similarities in precipitant and the use of either dog or cat DHX9 crystals to promote crystal growth, human DHX9 crystallized in space group *C*222_1_ with two molecules in the asymmetric unit. The crystal packing is highly similar in space groups *C*222_1_ and *P*4_3_2_1_2 and the crystal contacts are nearly identical. The second molecule in the human structure superimposes with a symmetry mate of cat DHX9 (Supplementary Fig. S4*a*) and similar packing interactions between human and cat DHX9 crystals were also observed in the extended space (Supplementary Fig. S4*b*). Equivalent results were seen in comparison with dog DHX9 (data not shown). Despite these parallels in crystal packing, data sets for human DHX9 crystals could not be processed in the higher symmetry space group *P*4_3_2_1_2.

### 3.2. Crystal structures of mammalian DHX9

The visible domains of the mammalian DHX9 structures form three well defined substructures consisting of (i) the MTAD and RecA1 domains, (ii) the RecA2 domain and (iii) the L2, HA2, OB and L3 domains (Fig. 1 ▸ *b*). The architecture of DHX9 is similar to that seen in *Drosophila* MLE (Prabu *et al.*, 2015 ▸), indicating that the overall structure has been conserved in higher species. However, structure determination of each orthologue revealed the absence of significant electron density for dsRBD2. Directed efforts to locate the dsRBD2 domain after model refinement resulted only in weak, disconnected density that did not allow confident placement of the domain; therefore, no residues of dsRBD2 were included in the final structures. To check whether *in situ* proteolysis of dsRBD2 had occurred, crystals of human DHX9 were dissolved and run on an SDS–PAGE gel (Supplementary Fig. S5). If dsRBD2 had been cleaved from the helicase domain, the remaining protein would have a molecular weight of approximately 100 kDa instead of 114.2 kDa, a size difference that would be visible by gel electrophoresis. However, the molecular weight of the protein from the crystal was identical to that of the purified human DHX9 used for crystallization; therefore, the dsRBD2 domain must be present but dynamic within the crystal lattice. For all three crystal forms, large solvent channels exist where electron density begins for the visible N-terminus of each chain. Presumably, these channels accommodate multiple poses of a flexible dsRBD2 domain, resulting in the absence of consequential electron density.

**Figure 2:**
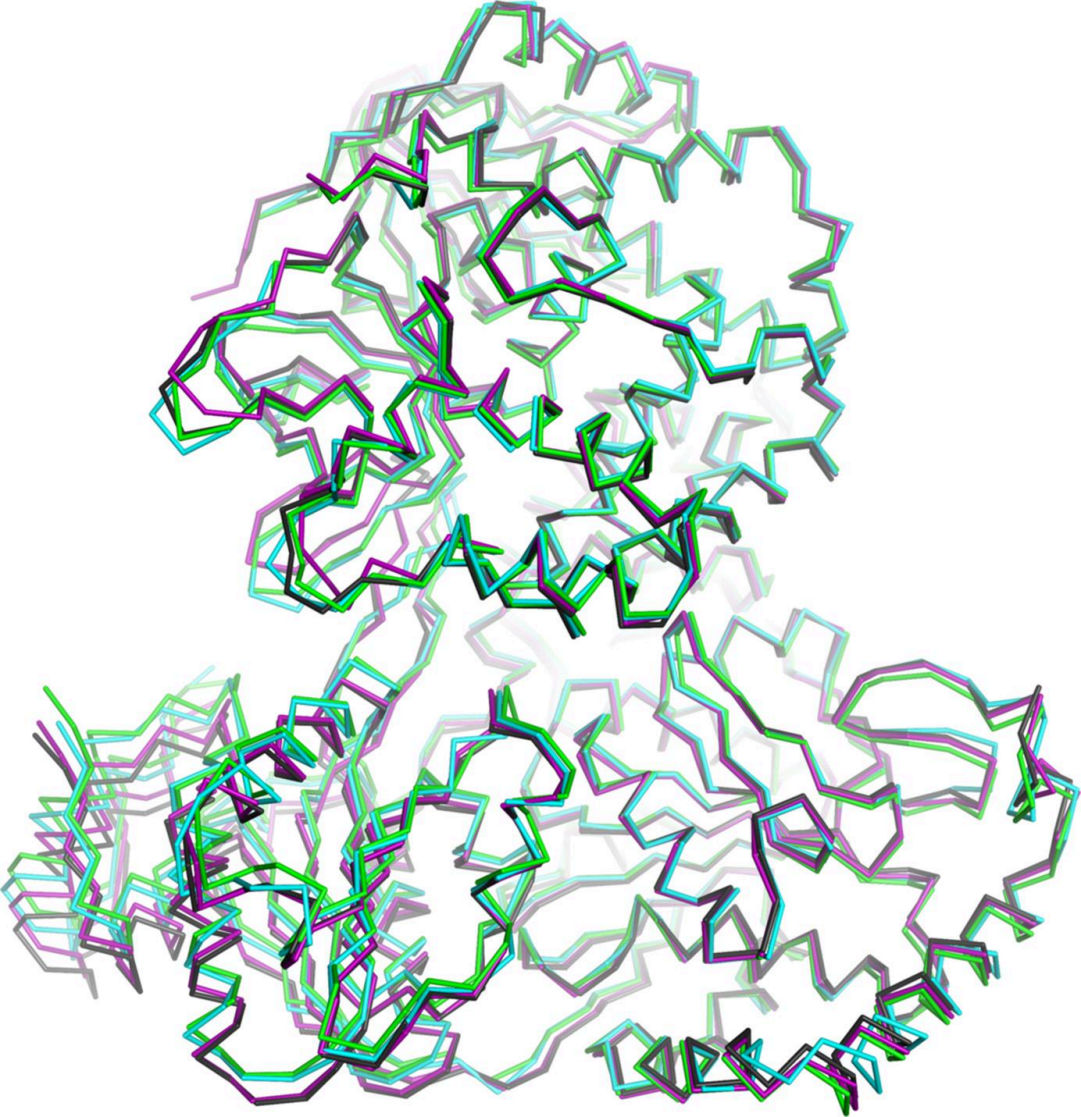
Mammalian DHX9 proteins share the same structural fold. Crystal structures of human DHX9 chain *A* (green) and chain *B* (black), dog DHX9 (cyan) and cat DHX9 (magenta) are superposed using all C^α^ atoms.

Superposition of the four independent protein chains of DHX9 in the three mammalian structures shows excellent conservation of the L2, MTAD, RecA1, HA2 and L3 domains between species (Fig. 2 ▸). The overall r.m.s.d. values for C^α^ atoms are less than 1.36 Å across all four chains of DHX9. The MTAD-RecA1 substructure in the human DHX9 structure reported here is highly homologous to the MTAD-RecA1 structure solved in the absence of other domains (PDB entry 3llm; Schütz *et al.*, 2010 ▸), with an r.m.s.d. of 0.85 Å. The RecA2 and OB-fold domains show the highest degree of conformational flexibility, with the largest differences in interdomain orientations observed between the two independent chains of the human protein (Fig. 2 ▸). These data indicate that the overall architecture of DHX9 is highly conserved within the mammalian species reported here, which is consistent with the high degree of sequence identity between the three proteins.

**Figure 3:**
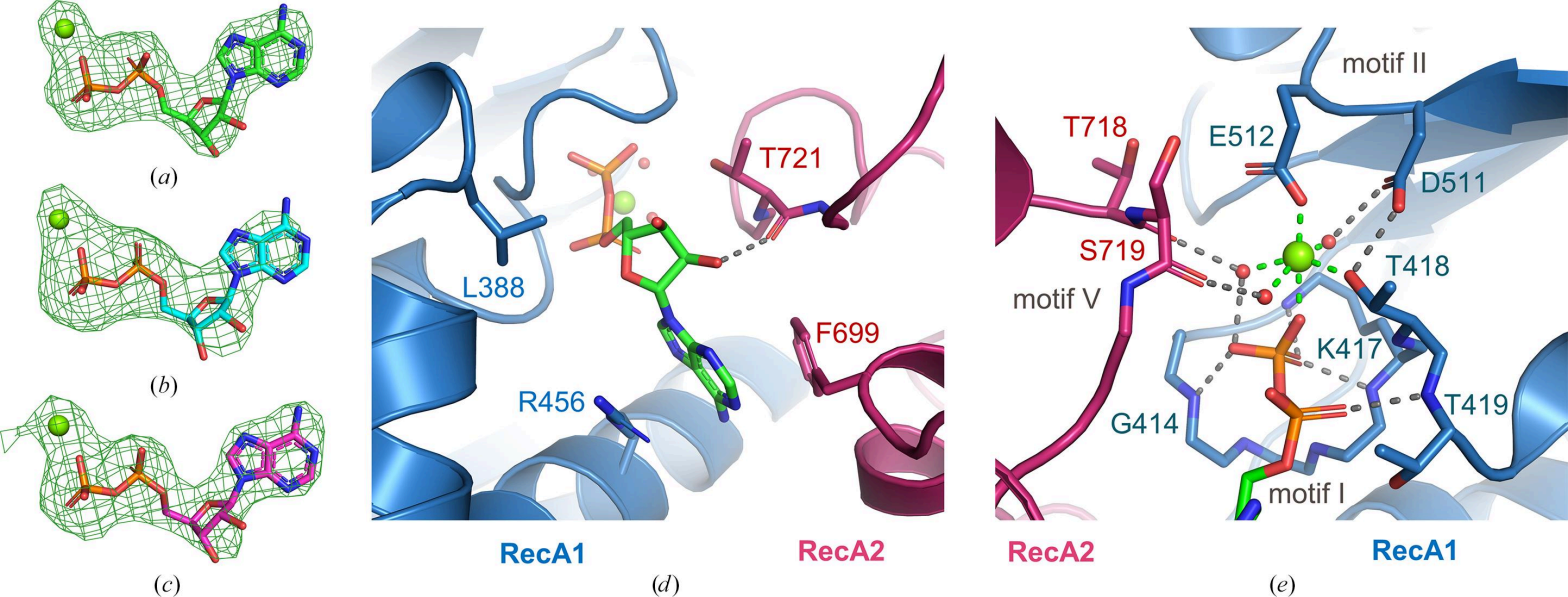
ADP- and metal-binding site of DHX9. (*a*, *b*, *c*) Electron-density omit maps (*F*_o_ − *F*_c_, 3.0σ) generated in the absence of ADP and Mg^2+^ for (*a*) human DHX9 chain *A* (green), (*b*) dog DHX9 (cyan) and (*c*) cat DHX9 (magenta). Mg^2+^ ions are shown as green spheres and ADP is shown in stick representation. (*d*) Interactions between the adenine and ribose of ADP (green stick representation) and human DHX9. Hydrogen bonds are shown as dashed lines; side chains that form hydrophobic or stacking interactions are labeled. (*e*) The coordination of Mg^2+^ (green sphere) is shown as green dashed lines. Hydrogen-bond interactions between ADP phosphate groups (stick representation), coordinated water molecules (red spheres) and DHX9 residues (stick representation) of human DHX9 are indicated as gray dashed lines. The DHX9 ribbon and side chains are colored as in Fig. 1 ▸.

The interface of the RecA domains forms the nucleotide-binding pocket. For all three mammalian proteins, the density for ADP and the coordinated Mg^2+^ ion are well defined in the omit map generated by refinement in the absence of ADP and Mg^2+^ (Figs. 3 ▸ *a*–3 ▸ *c*). ADP and Mg^2+^ bind to DHX9 through multiple interactions with both the RecA1 and RecA2 domains (Figs. 3 ▸ *d* and 3 ▸ *e*), which is generally consistent with other structures of ADP-bound DE*x*H-box helicases (Felisberto-Rodrigues *et al.*, 2019 ▸; He *et al.*, 2010 ▸; Murakami *et al.*, 2017 ▸; Chen *et al.*, 2018 ▸; Schmitt *et al.*, 2018 ▸; Walbott *et al.*, 2010 ▸; Tauchert *et al.*, 2017 ▸). The adenine base forms a cation–π interaction with Arg456 in RecA1 (the amino-acid numbering refers to human DHX9) and a π–π stacking interaction with Phe699 in RecA2 (Fig. 3 ▸ *d*). No hydrogen-bond interactions with the protein were observed for the purine ring. The ribose ring forms hydrophobic interactions with the side chains of Leu388 in RecA1 and Thr721 in RecA2, while the ribose 2′-OH makes a hydrogen bond to the backbone carbonyl O atom of Thr721 (Fig. 3 ▸ *d*). Nucleotide-binding motifs I, II and V form the expected contacts with the phosphate groups of ADP and the coordinated Mg^2+^ (Fig. 3 ▸ *e*); Mg^2+^ interacts with the side chains of Thr418 (motif I), Glu512 (motif II), three water molecules and a β-phosphate O atom from ADP in coordination patterns typical for the ion (Case *et al.*, 2020 ▸). Mg^2+^-coordinated water molecules are further stabilized by interactions with Asp511 (motif II) and the carbonyl O atoms of Thr718 and Ser719 (motif V). The β-phosphate O atoms that are not involved in Mg^2+^ coordination engage in extensive hydrogen-bond networks with residues in motif I, which include the backbone amides of Gly414, Thr418 and Thr419, as well as the side chain of Lys417. The Mg^2+^ and nucleotide interactions described for human DHX9 are also conserved in the dog and cat DHX9 structures (Supplementary Fig. S6).

### 3.3. Comparison of human DHX9 with substrate-bound MLE

**Figure 4:**
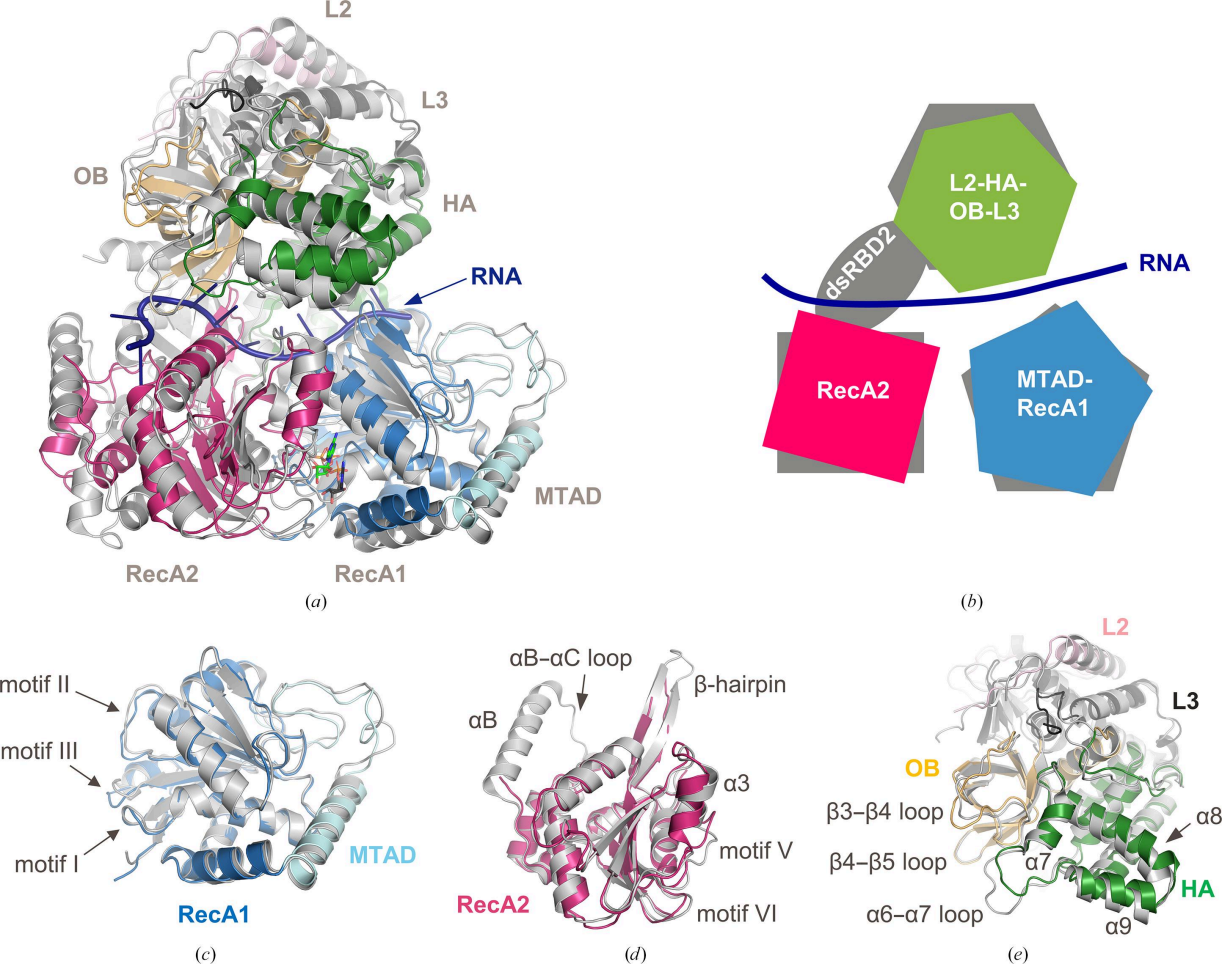
Superposition of DHX9 and MLE. The color scheme of the DHX9 domains is identical to that in Fig. 1 ▸; MLE is colored gray. (*a*) Global superposition of DHX9 and MLE structures generated using all C^α^ atoms (dsRBD2 is not included for MLE). ADP (DHX9-bound) and ADP–AlF_4_ (MLE-bound) are shown in in green and gray stick representations, respectively; RNA (bound to MLE) is shown in a dark blue cartoon representation. (*b*) Cartoon representation of the global conformational differences seen between domains in the superposition of DHX9 (color) and MLE/RNA (gray/dark blue); dsRBD2 is included for MLE. Superpositions of individual substructures are shown: (*c*) MTAD-RecA1, (*d*) RecA2 and (*e*) L2-HA-OB-L3.

Full-length human DHX9 shares 50.6% sequence identity with the *Drosophila* DHX9 orthologue (Supplementary Fig. S2). However, structural alignment using all visible domains of DHX9 with MLE reveals noteworthy differences in the global structure of these orthologues, despite their highly similar overall architecture (Figs. 4 ▸ *a* and 4 ▸ *b*). The r.m.s.d. between chain *A* of human DHX9 and chain *A* of MLE is 4.05 Å. However, higher structural conservation is seen for the individual substructures when compared in isolation. The MTAD-RecA1 substructures have the highest structural similarity, with an r.m.s.d. value of 1.10 Å (Fig. 4 ▸ *c*), while the RecA2 domains have a slightly higher r.m.s.d. value of 2.80 Å (Fig. 4 ▸ *d*). The substructure composed of the L2-HA2-OB-L3 domains has the highest r.m.s.d. value of 3.24 Å (Fig. 4 ▸ *e*). Given these observations, a closer examination of the source of the structural differences that are seen between the DHX9 and MLE proteins is warranted.

Global structural changes as well as localized conformational differences between DHX9 and MLE are driven by three distinct, yet interconnected, features: (i) the intramolecular interactions in the presence or absence of dsRBD2, (ii) the binding of RNA substrate (or lack thereof) and (iii) the identity of the bound nucleotide. The DHX9 structures were solved with the product of the hydrolysis reaction, ADP, in the absence of RNA. Hence, DHX9 represents an inactive state of the enzyme. In contrast, the *Drosophila* MLE structure, which was solved with RNA and the transition-state mimetic ADP–AlF_4_, represents one of many conformations of the enzyme found during the catalytic cycle and for the purposes of subsequent analyses will be considered to be an active-state structure. Comparison of these distinct structures representing different catalytic states provides insights into how substrate binding and the interactions of the dsRBD2 domain induce localized conformational changes and global domain orientation shifts in the DHX9/MLE protein family that are required for enzyme activity. To enable detailed analysis of structural changes, human DHX9 chain *A* (hereafter referred to as DHX9) and MLE chain *A* were aligned using their RecA1 domains (Supplementary Fig. S7 and Supplementary Videos S1 and S2). This alignment will be used throughout the remainder of this section unless otherwise indicated.

#### 3.3.1. dsRBD2

**Figure 5:**
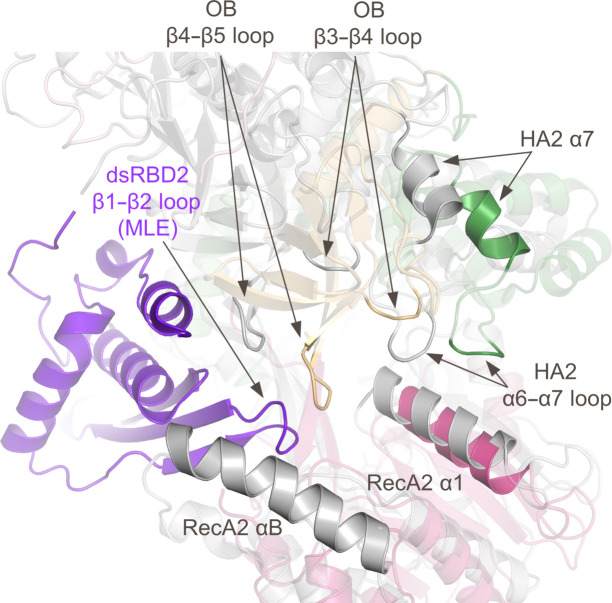
Superposition of DHX9 and MLE shows structural changes due to the absence of dsRBD2 in DHX9. The color scheme of the DHX9 domains is identical to that in Fig. 1 ▸. MLE is shown in gray, except for dsRBD2 which is shown in purple. Superposition was generated by aligning the RecA1 domains of both proteins (the RecA1 domains are not shown).

As previously noted, dsRBD2 is not visible in the structures of mammalian DHX9 due to flexibility of the domain within the crystal lattice. The absence of dsRBD2 has a profound effect on the structure of the region of DHX9 that would engage with the domain when compared with MLE. Without stabilizing contributions from the dsRBD2 domain, structural features of RecA2 including helix αB, the αB–αC loop and the β-hairpin are disordered in the DHX9 structure (Fig. 4 ▸ *c*). Additionally, rotation of the OB, HA2 and RecA2 domains occurs in the absence of the dsRBD2 domain in DHX9 when compared with MLE, significantly changing the topography in this region (Fig. 5 ▸). dsRBD2 and its extensive interactions with other domains form important features for RNA binding in MLE and stabilize the active enzyme conformation (Prabu *et al.*, 2015 ▸). In DHX9, disorder of dsRBD2 also contributes to structural changes that impact the RNA-binding channel and nucleotide-binding site, as further outlined below.

#### 3.3.2. RNA channel

The inactive conformation of DHX9 lacks an open, continuous channel through which RNA could bind. Therefore, it is unsurprising that there are significant differences between the DHX9 and RNA-bound MLE structures in and near the RNA-binding channel. In MLE, the RNA-binding channel, from 5′ to 3′, is formed by the dsRBD2, RecA2, OB, HA, RecA1 and MTAD domains. DExH-box helicases typically require the presence of a 3′ overhang for unwinding activity (Jankowsky, 2011 ▸; Ozgur *et al.*, 2015 ▸; Pyle, 2008 ▸) and unwind in the 3′-to-5′ direction. Therefore, in the analysis below, the region where the 3′ end of RNA was visible in MLE will be referred to as the ‘exit’ site and the area where the 5′ RNA end was observed is designated as the ‘entrance’ site.

**Figure 6:**
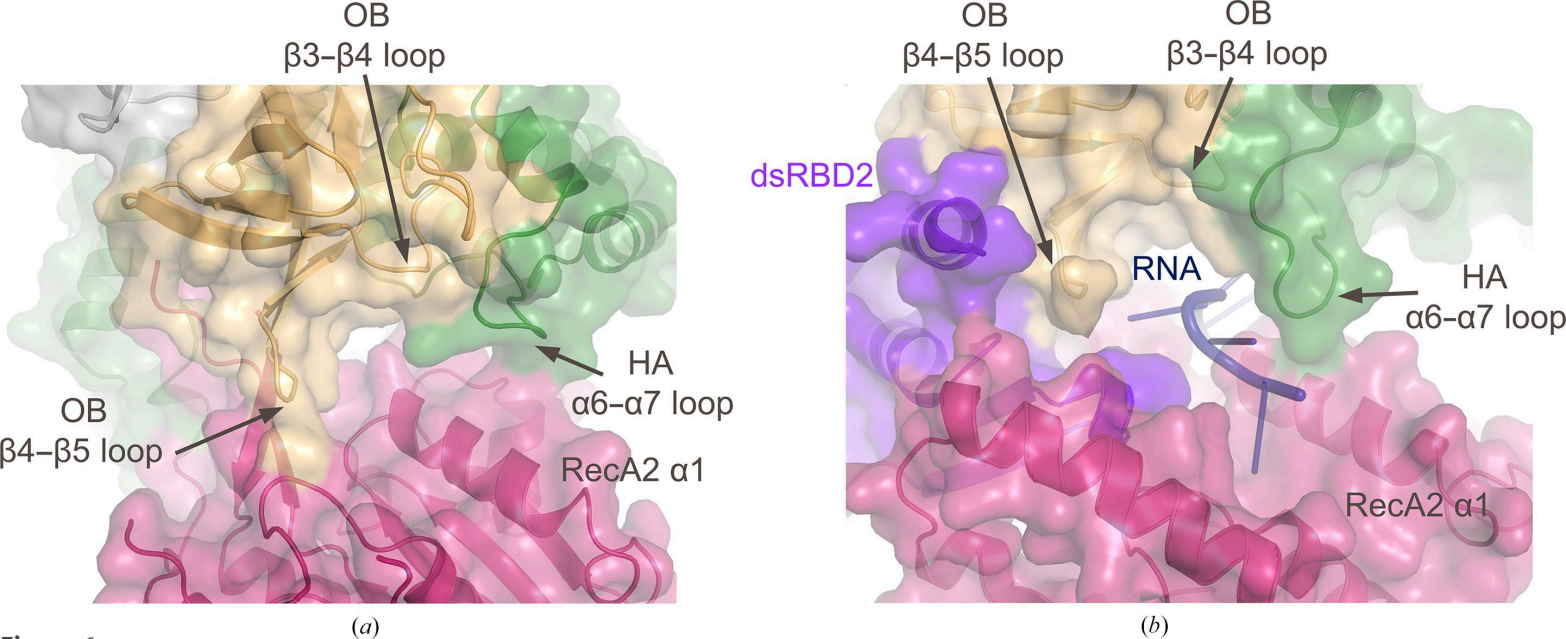
Comparison of the RNA channel entrance in DHX9 and MLE. (*a*) Composite cartoon and surface representation of DHX9. The color scheme of DHX9 is identical to that in Fig. 1 ▸. (*b*) Composite cartoon and surface representation of MLE. The color scheme used for the DHX9 domain structure has been applied to MLE. The dsRBD2 domain is shown in purple and RNA is shown in dark blue cartoon representation.

Due to the dynamics of dsRBD2 in DHX9, only the OB, HA2 and RecA2 domains are visible at the RNA entrance site, resulting in a distinct conformation. Visible residues in RecA2 in this region (α1) show slight shifts in position between DHX9 and MLE, while significant conformational changes in the OB domain result in a constricted entry point in DHX9 relative to MLE (Fig. 6 ▸). In the presence of RNA, OB loops β3–β4 and β4–β5, as well as HA2 loop α6–α7, form key portions of the RNA channel (Prabu *et al.*, 2015 ▸). In DHX9, the β3–β4 and β4–β5 loops in the OB domain shift by ∼9 and ∼10 Å, respectively. As a result, OB loop β4–β5 occupies a region of space in DHX9 that is filled by the β1–β2 loop of dsRBD2 and RNA bases in MLE (Fig. 5 ▸). Overlays of the individual substructure containing the HA2 domain and OB-fold (Fig. 4 ▸ *e*) show that conformational changes within the OB domain itself are minor, with the β3–β4 and β4–β5 loops undergoing local conformational shifts of 1–3.4 and <1 Å, respectively. Therefore, the large 9–10 Å shifts are largely due to dsRBD2-induced changes in global domain orientation. Presumably, the OB domain moves to enable RNA binding and accommodate association of dsRBD2 during the catalytic cycle. Structural changes in the OB-fold also impact the HA2 domain. Helices α7 and loop α6–α7 in HA2 both move ∼10 Å to facilitate rearrangement of β3–β4 in the OB loop (Fig. 5 ▸). Direct comparison of the OB/HA2 substructure in DHX9 and MLE shows significant local rearrangement (∼6 Å) of the long α6–α7 loop but a similar position for the α7 helix (Fig. 4 ▸ *e*). Therefore, while both the HA2 and OB domains undergo global motions that result in remodeling of the RNA channel entrance, local loop rearrangements play a larger part in the structural changes of the HA2 domain than is seen within the OB domain.

**Figure 7:**
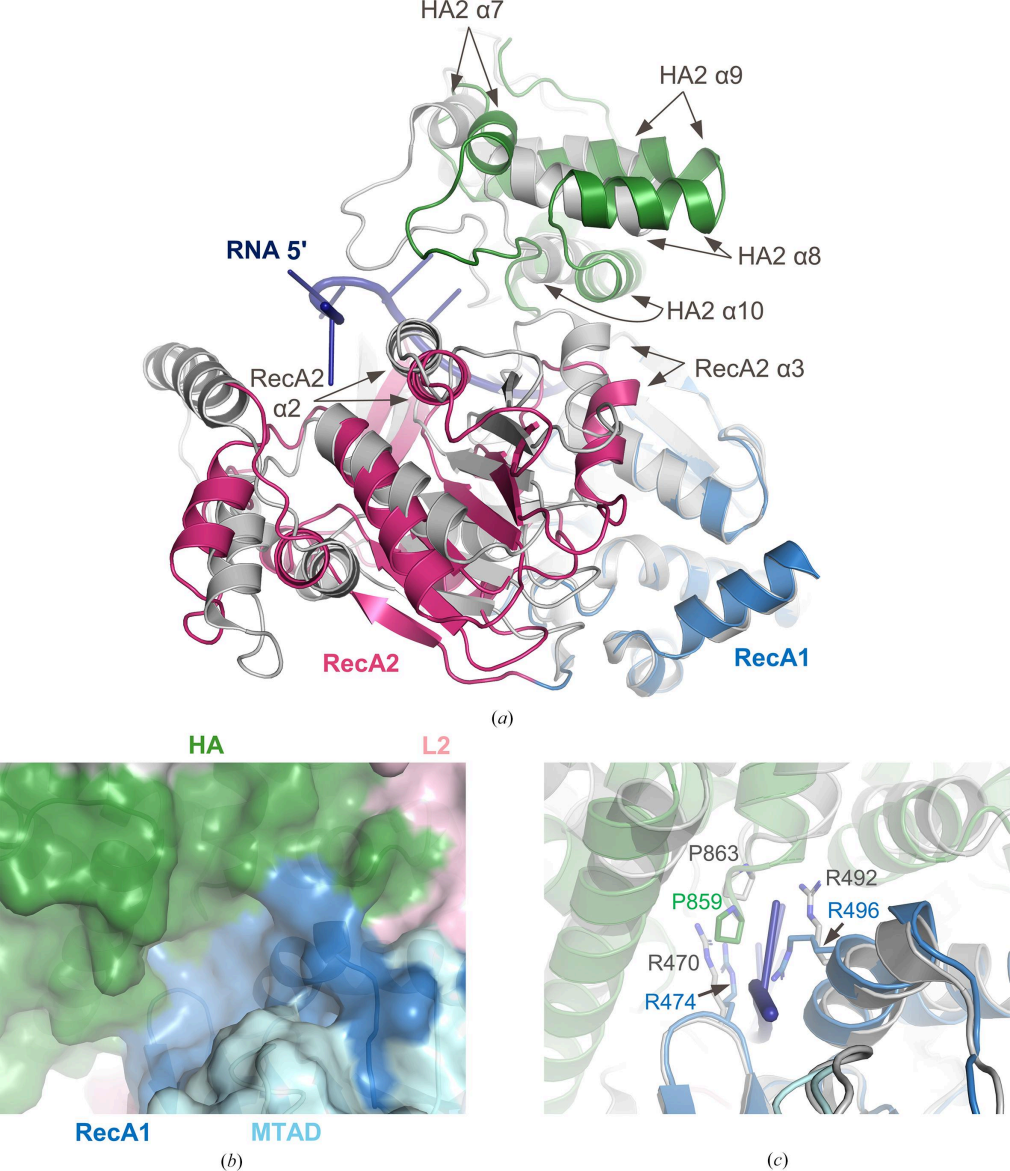
Structural changes at the exit site of the RNA channel. The color scheme for DHX9 is identical to that in Fig. 1 ▸; MLE is shown in gray and RNA is in dark blue. (*a*) Superposition of DHX9 and MLE shows structural changes of secondary structures that impact the RNA channel. (*b*) A composite cartoon and surface representation of DHX9 shows structural changes that result in complete closure of the RNA exit site. (*c*) Side-chain movements in RecA1 and domain movements of the secondary structure of the HA domain impact the RNA exit site. Residues are shown in stick representation.

The global rearrangement of the RecA2 domain and OB/HA2 substructure described previously due to the absence of dsRBD2 also impacts other portions of the RNA-binding channel. Shifts in HA2 helix α10 and RecA2 helices α2 and α3 dramatically change the interface between the substructures where RNA binds in MLE (Fig. 7 ▸ *a*). Structural changes also impact the RNA exit site, resulting in a complete collapse of the channel in this region of the protein (Fig. 7 ▸ *b*). Side-chain movements of Arg474 and Arg496 of RecA1, as well as a 3 Å movement of the α4–α5 loop in HA2, including Pro859, fill the exit site in the absence of RNA (Fig. 7 ▸ *c*). Given the high degree of structural similarity in the RecA1 domains between MLE and DHX9, it is not surprising that the arginine side-chain movements that are described are local changes, while the movements in the α4–α5 loop in HA2 are long-range structural changes that result from the absence of dsRBD2. The changes seen throughout the interfaces between the DHX9 substructures further emphasize the impact of a disordered dsRBD2 on the RNA-binding site.

#### 3.3.3. Nucleotide-binding site

**Figure 8:**
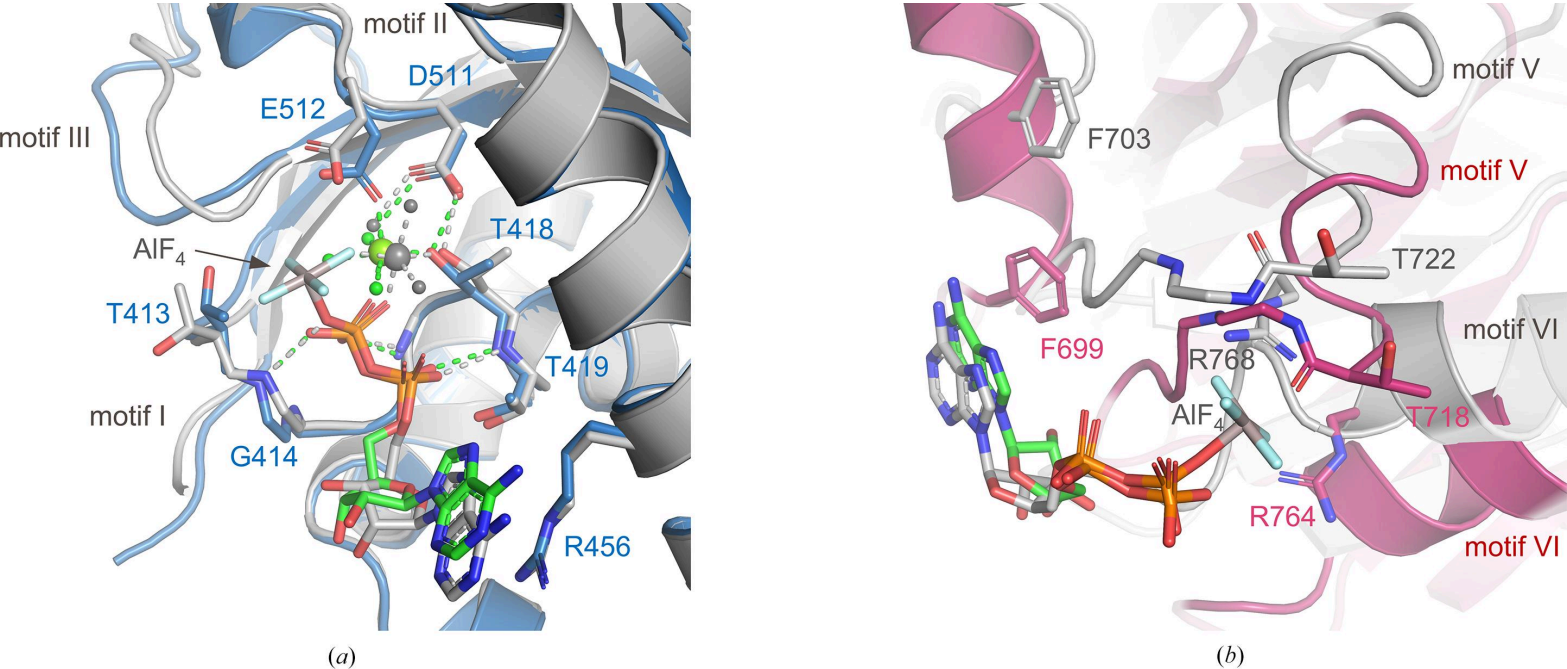
Comparison of interactions at the nucleotide-binding sites of DHX9 and MLE. DHX9 and MLE structures are superposed relative to the RecA1 domain and are colored as in Fig. 4 ▸. Motif and residue labels are shown in blue or dark pink for DHX9 and in black for MLE. ADP in DHX9 and ADP–AlF_4_ in MLE are colored green and gray, respectively. (*a*) Interactions at the RecA1 interface. Mg^2+^ (large spheres), coordinated waters (small spheres) and their coordination (dashed lines) in DHX9 and MLE are colored to match the associated nucleotide. Only DHX9 residues are labeled for simplicity. (*b*) Interactions at the RecA2 interface are shown.

In these analyses, DHX9 and MLE were aligned by their RecA1 domain. Therefore, similarities in the RecA1-mediated binding features for ADP and ADP–AlF_4_ that are bound to DHX9 and MLE, respectively, are obvious, and any differences are due to localized conformational changes. The adenine base maintains a π-stack with a conserved arginine residue (Arg456) despite slight shifts in the position of the nucleotide base and ribose ring (Fig. 8 ▸ *a*). The α- and β-phosphates are well superposed in the two structures and the hydrogen bonds to backbone atoms are well conserved. Despite a slight shift in the position of the metal, four of the six coordinating ligands of Mg^2+^ are maintained: the β-phosphate, Thr418 in motif I and two water molecules. However, in DHX9 the side chain of Glu512 in motif II displaces a coordinating water molecule seen in MLE and another water molecule fills the coordination site occupied by the AlF_4_ moiety. The removal of AlF_4_ also causes localized conformational changes, including rotation of the side chain of Thr413 in motif I.

As the nucleotide-binding site is positioned at the interface of the RecA1 and RecA2 domains, the global movements of RecA2 described previously also dramatically impact the interactions formed by RecA2 with ADP and Mg^2+^, breaking many interactions that are seen in the MLE structure and creating new interactions that are only seen in the inactive conformation of DHX9. The previously described π-stacking interaction between the adenine base and Phe699 (Fig. 3 ▸ *d*) is formed due to rotation of the RecA2 domain relative to RecA1 and is absent in MLE (Fig. 8 ▸ *b*). This rotation also shifts key residues far from ADP that engage in important nucleotide interactions in the MLE structure. These include the side chain of Arg764 in motif IV (Arg768 in MLE), which interacts with the AlF_4_ moiety in MLE; Arg764 shifts ∼8 Å in DHX9 and can no longer engage with the nucleotide (Fig. 8 ▸ *b*). In contrast to these large shifts in position relative to RecA1, residues in motif V of RecA2 undergo localized conformational change in order to maintain their proximity to RecA1 and interact with ADP in DHX9 (Supplementary Video S2). For example, the backbone amide of Thr718 reverses its orientation (Fig. 8 ▸ *b*), allowing the backbone carbonyl to form a hydrogen bond to the metal-coordinating water molecule in DHX9 that occupies the position of the γ-phosphate moiety in MLE (Fig. 3 ▸ *e*). Interestingly, evaluation of these structural differences in the context of the global protein structure reveals that the changes in the positions of Arg764 and Phe699 are solely the result of RecA2 domain rotation taking place in concert with dsRBD2 binding, as no conformational rearrangements of secondary-structure elements or side chains occur. Motif V, on the other hand, undergoes significant local conformational rearrangement to maintain the described binding interactions as the RecA2 domain rotates to accommodate the dsRBD2 domain. In other words, local changes in motif V are a way to compensate for the global motion of RecA2 in order to maintain engagement with the nucleotide-binding site, metal ion and RecA1 domain.

## 4. Discussion

DHX9 is a target of therapeutic interest in oncology due to its critical role in maintaining genomic stability. Structure-based design is a valuable tool for any drug-discovery program; however, an experimental structure of the target of interest is greatly preferred and crystal structures of the human protein are desirable when targeting human disease. Although the structure of the *Drosophila* DHX9 orthologue MLE was known, it would be of limited use for drug design due to the relatively low sequence identity (50.61%) between MLE and human DHX9. However, the structure of MLE was valuable in efforts to crystallize DHX9; appropriate N- and C-terminal truncations in MLE were identified and directly applied to construct design for mammalian DHX9 ortho­logues, and MLE was successfully used for molecular replace­ment.

Despite the identification of a suitable construct, human DHX9 was initially recalcitrant to crystallization. There are numerous protein-engineering approaches to stabilize proteins and promote crystallization, including surface-entropy reduction, surface mutagenesis disulfide engineering and chemical modification (Deller *et al.*, 2016 ▸; Derewenda & Vekilov, 2006 ▸). However, altering the protein surface or reducing the conformational flexibility of a protein to promote crystallization could limit the utility of the structure as a tool for drug-discovery efforts if the residues affected are involved in compound binding or if the conformation of the protein required for compound binding is no longer accessible. The use of species orthologues in crystallization screening allows the exploration of alternative sequences that may crystallize with a minimal impact on the overall structure or protein activity. Therefore, the DHX9 crystallization campaign focused on species orthologues with ∼95% identity. Cat and dog DHX9 had 22 and 23 residue differences, respectively, from the human protein construct and many of the changes were predicted to be in surface residues. It is unknown whether the relatively small increases in melting temperature compared with human DHX9 seen for dog DHX9 (1.3°C) and cat DHX9 (3.2°C) contributed to their ability to form crystals; studies investigating thermal stability and crystallization indicate that the crystallization potential does not statistically increase for temperatures above ∼45°C (Dupeux *et al.*, 2011 ▸). Regardless, orthologue screening was ultimately successful in enabling the structure determination of mammalian DHX9. Additionally, the orthologue crystals were successfully used as seeds for the human protein, enabling crystallization and subsequent structure determination. Although this is not a new or novel technique (McPherson & Gavira, 2014 ▸), it serves as a reminder that this approach is a viable option that can be explored for other human proteins that are refractory to crystallization. Additionally, cat and dog DHX9 provide additional crystallization systems that could enable iterative structure-based drug-design efforts with validated compounds from DHX9-focused hit-finding efforts, assuming that the compounds bind to the orthologue proteins.

The structures of the individual globular domains of mammalian DHX9 proteins and *Drosophila* MLE are well conserved; however, global rotation of domains and localized conformational changes are seen in structural comparisons. As DHX9 bound to ADP represents an inactive state of the helicase, while MLE bound to RNA and a transition-state analogue represents an active state, structural variation is expected. The nature and scope of the structural differences seen between DHX9 and MLE appear to be consistent with other DE*x*H-box proteins in that smaller structural changes (∼5 Å) between core helicase domains are seen for different conformational states (Ozgur *et al.*, 2015 ▸). Indeed, the rotation of globular domains in the presence of varying substrates was also seen for the DE*x*H-box helicase DHX36 (Srinivasan *et al.*, 2020 ▸). However, the absence of dsRBD2 in the mammalian DHX9 structures due to conformational flexibility within the protein crystals was unexpected. Intriguingly, recent structural studies of MLE using cryo-EM revealed that apo and ADP-AlF4-bound MLE in the absence of RNA lacked density for dsRBD2 and in addition indicated that binding of the dsRBD2 domain to the core helicase may be linked to RNA channel opening (Jagtap *et al.*, 2022 ▸). The analysis and conclusions presented in this report, that the absence of dsRBD2 binding to the core helicase results in large conformational changes that impact both the RNA-binding and nucleotide-binding sites in addition to local conformational changes at the site of dsRBD2 binding, would appear to be consistent with those of Jagtap and coworkers. These additional structures of MLE determined by Jagtap and coworkers are not yet publicly available; therefore, a direct structural comparison with ADP-bound DHX9 could not be performed. However, continued biochemical, biophysical and structural studies of the DHX9 enzyme and species orthologues will provide additional understanding of the impact of dsRBD domains as well as nucleotide and RNA binding on helicase conformational changes that result in the functional unwinding activity of the enzymes.

The structures of DHX9 bound to ADP presented here are the first mammalian structures of this helicase to be solved. Comparison of this inactive conformation with structures of *Drosophila* MLE provide important information regarding the structural states sampled during substrate unwinding for this DE*x*H-box helicase. In addition, these structures may provide a useful tool to enable structure-based design for future drug-discovery efforts.

## Supplementary Material

PDB reference: human DHX9 with ADP, 8szp

PDB reference: dog DHX9 with ADP, 8szr

PDB reference: cat DHX9 with ADP, 8szq

Supplementary videos in PowerPoint slide format. DOI: 10.1107/S2059798323007611/ag5044sup1.pptx

Supplementary Figures/. DOI: 10.1107/S2059798323007611/ag5044sup2.pdf

## Figures and Tables

**Table 1:** Crystallization conditions

Protein	Human DHX9 (150–1150)	Dog DHX9 (151–1151)	Cat DHX9 (151–1151)
Method	Hanging drop	Hanging drop	Hanging drop
Plate type	24-well	24-well	24-well
Temperature (K)	291	291	291
Protein concentration (mg ml^−1^)	8	8	8
Buffer composition of protein solution	20 m*M* HEPES, 300 m*M* NaCl, 2 m*M* TCEP pH 7.5, 5 m*M* ADP, 50 m*M* MgCl_2_	20 m*M* HEPES, 300 m*M* NaCl, 2 m*M* TCEP pH 7.5, 5 m*M* ADP, 50 m*M* MgCl_2_	20 m*M* HEPES, 300 m*M* NaCl, 2 m*M* TCEP pH 7.5, 5 m*M* ADP, 50 m*M* MgCl_2_
Composition of reservoir solution	200 m*M* lithium sulfate, 100 m*M* MES pH 6, 20%(*w*/*v*) PEG 4000	200 m*M* lithium sulfate, 100 m*M* MES pH 6, 20%(*w*/*v*) PEG 4000	150 m*M* ammonium sulfate, 100 m*M* Tris pH 8, 19%(*w*/*v*) PEG 4000
Volumes of protein/reservoir in crystallization drop (µl)	1.5/1.5	1.5/1.5	1.5/1.5
Volume of reservoir (µl)	500	500	500

**Table 2:** Data-collection and processing statistics Values in parentheses are for the outer shell.

Protein	Human DHX9 (150–1150)	Dog DHX9 (151–1151)	Cat DHX9 (151–1151)
Diffraction source	I03, Diamond Light Source	MX2, Australian Synchrotron	MX2, Australian Synchrotron
Wavelength (Å)	0.97624	0.95364	0.95364
Temperature (K)	100	100	100
Detector	EIGER2 XE 16M	EIGER X 16M	EIGER X 16M
Crystal-to-detector distance (mm)	376.565	379.983	379.983
Rotation range per image (°)	0.1	0.1	0.1
Total rotation range (°)	360	360	360
Exposure time per image (s)	0.006	0.010	0.010
Space group	*C*222_1_	*P*4_3_2_1_2	*P*4_3_2_1_2
*a*, *b*, *c* (Å)	119.10, 122.26, 349.18	85.29, 85.29, 352.10	86.01, 86.01, 348.85
α, β, γ (°)	90, 90, 90	90, 90, 90	90, 90, 90
Mosaicity (°)	0.082	0.050	0.059
Resolution range (Å)	61.13–2.62 (2.67–2.62)	48.35–2.97 (3.15–2.97)	48.17–2.71 (2.83–2.71)
Total no. of reflections	980269	734995	959448
No. of unique reflections	76541	27977	36693
Completeness (%)	99.7 (99.4)	100.0 (99.9)	99.8 (98.2)
Multiplicity	12.8 (14.0)	26.3 (25.7)	26.1 (25.5)
〈*I*/σ(*I*)〉	21.8 (2.2)	33.0 (3.2)	23.1 (2.9)
*R*_r.i.m._ †	0.064 (1.450)	0.067 (1.327)	0.129 (1.549)
CC_1/2_	1.000 (0.815)	1.000 (0.902)	1.000 (0.791)
Overall *B* factor from Wilson plot (Å^2^)	71.0	97.1	57.5

† Estimated *R*_r.i.m._ = *R*_merge_[*N*/(*N* − 1)]^1/2^, where *N* is the data multiplicity.

**Table 3:** Structure-determination and refinement statistics Values in parentheses are for the outer shell.

Protein	Human DHX9 (150–1150)	Dog DHX9 (151–1151)	Cat DHX9 (151–1151)
Resolution range (Å)	59.62–2.62 (2.69–2.62)	48.39–2.97 (3.05–2.97)	48.22–2.71 (2.78–2.71)
Completeness (%)	99.6 (99.0)	99.9 (99.5)	99.7 (96.9)
σ Cutoff	None	None	None
No. of reflections, working set	68960 (5267)	25221 (1377)	33046 (1822)
No. of reflections, test set	3769 (283)	1927 (95)	2461 (124)
Final *R*_cryst_	0.219 (0.348)	0.243 (0.368)	0.226 (0.337)
Final *R*_free_	0.274 (0.378)	0.296 (0.477)	0.274 (0.397)
Cruickshank DPI	0.527	—	0.632
No. of non-H atoms			
Protein	13594	6785	6831
Ion (Mg^2+^)	2	1	1
Ligand (ADP)	54	27	27
Water	134	5	94
Total	13893	6848	7013
R.m.s. deviations			
Bond lengths (Å)	0.0027	0.0024	0.0019
Angles (°)	0.8768	0.8135	0.7083
Average *B* factors (Å^2^)			
Protein	90.1	122.3	73.2
Ion (Mg^2+^)	69.5	90.9	57.6
Ligand (ADP)	86.3	119.0	64.3
Water	69.5	84.5	57.1
Ramachandran plot			
Most favored (%)	95	93	96
Allowed (%)	5	7	4
