# Supplementary material for: Crystal structures of the DExH-box RNA helicase DHX9
Source: Acta Crystallogr D Struct Biol. 2023 Oct 20;79(Pt 11):980–91. doi: 10.1107/S2059798323007611 (PMC10619421; doi:10.1107/S2059798323007611)
Supplement: Supplementary file 1 [file d-79-00980-sup1.pptx]

## Slide 1
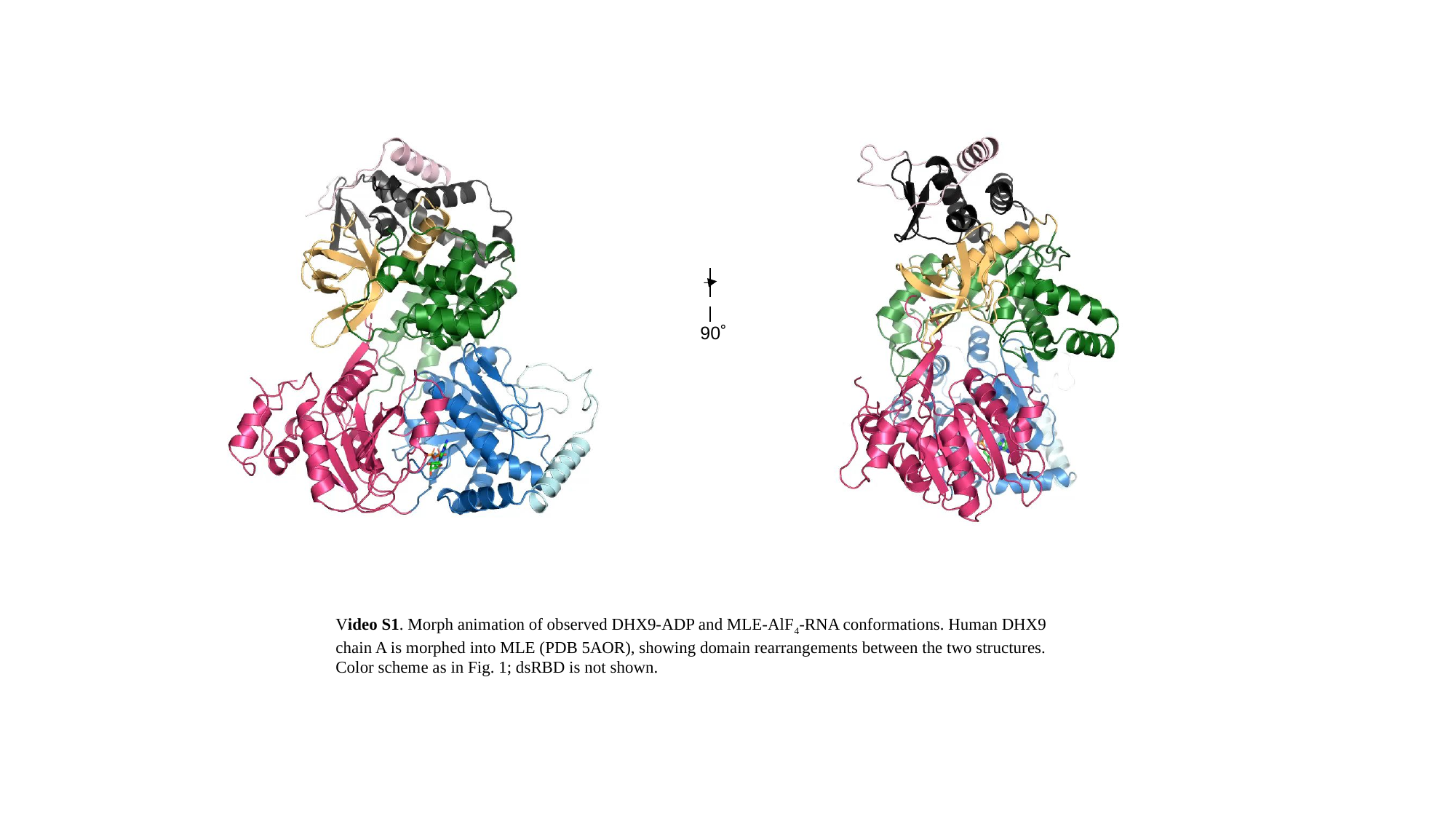

90˚
Video S1. Morph animation of observed DHX9-ADP and MLE-AlF4-RNA conformations. Human DHX9 chain A is morphed into MLE (PDB 5AOR), showing domain rearrangements between the two structures. Color scheme as in Fig. 1; dsRBD is not shown.

## Slide 2
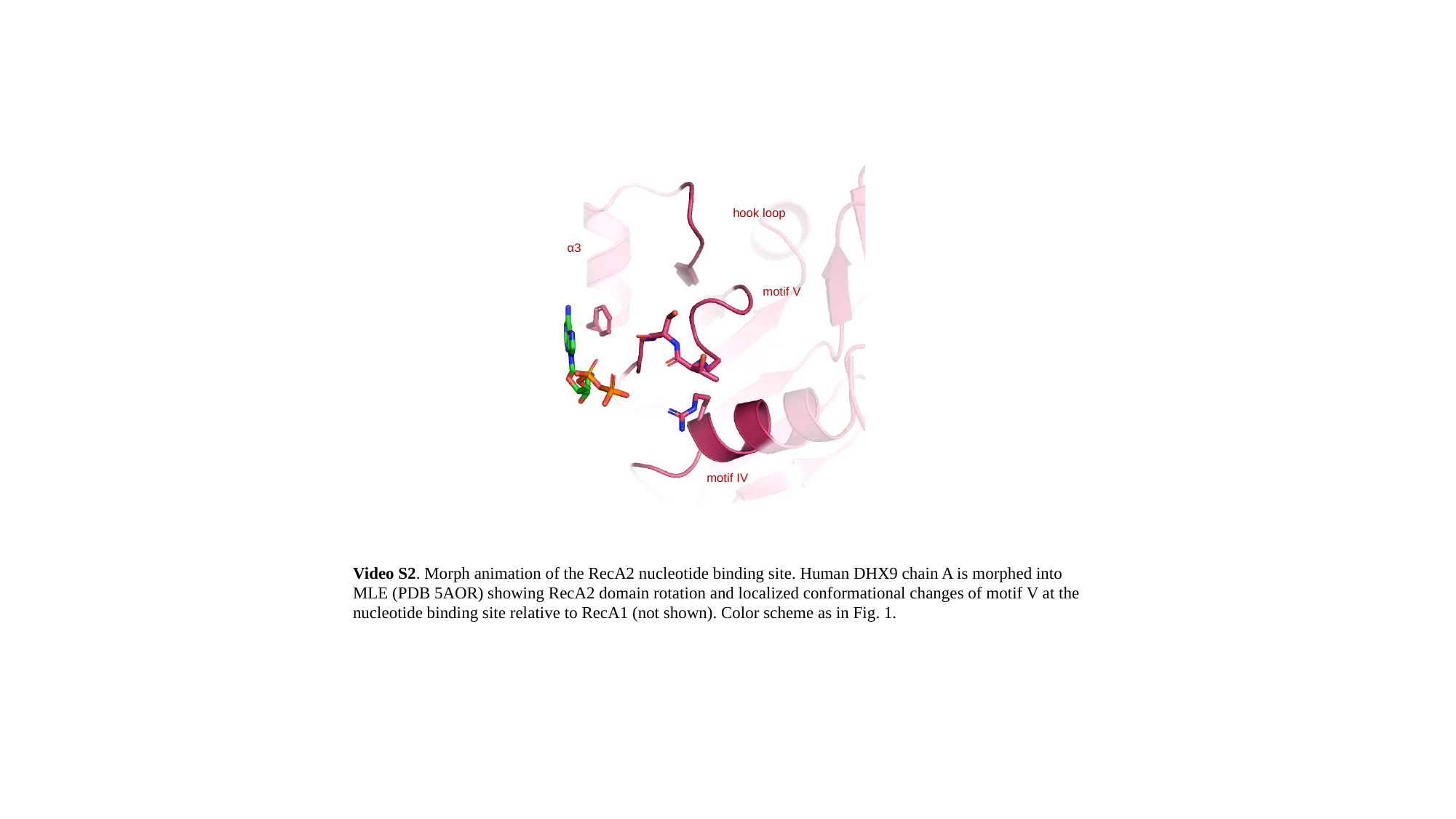

hook loop
α3
motif V
motif IV
Video S2. Morph animation of the RecA2 nucleotide binding site. Human DHX9 chain A is morphed into MLE (PDB 5AOR) showing RecA2 domain rotation and localized conformational changes of motif V at the nucleotide binding site relative to RecA1 (not shown). Color scheme as in Fig. 1.
