# Supplementary material for: Crystal structures of the DExH-box RNA helicase DHX9
Source: Acta Crystallogr D Struct Biol. 2023 Oct 20;79(Pt 11):980–91. doi: 10.1107/S2059798323007611 (PMC10619421; doi:10.1107/S2059798323007611)
Supplement: Supplementary file 2 [file d-79-00980-sup2.pdf]

STRUCTURAL BIOLOGY

Volume 79 (2023)

Supporting information for article:

Crystal structures of the DE*x*H-box RNA helicase DHX9

Young-Tae Lee, E. Allen Sickmier, Simina Grigoriu, Jennifer Castro and P. Ann Boriack-Sjodin

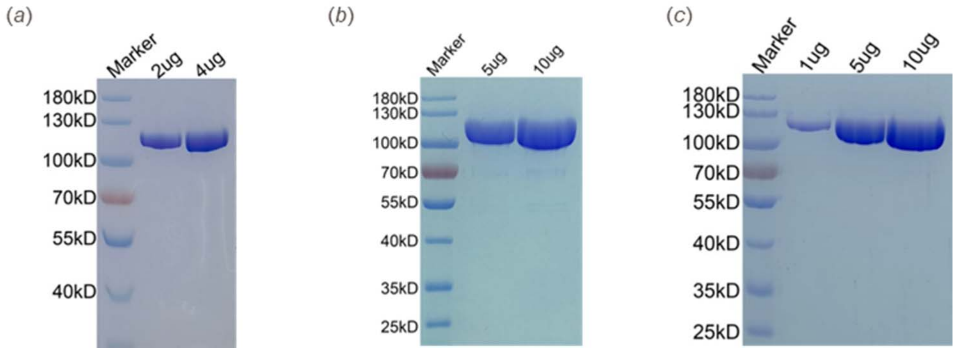

Figure S1 SDS-PAGE of purified mammalian DHX9 proteins used for crystallization. (*a*) Human DHX9 (residues 150-1150, 114.2 kDa), (*b*) Dog DHX9 (residues 151-1151, 114.4 kDa), (*c*) cat DHX9 (residues 151-1151, 114.5 kDa).

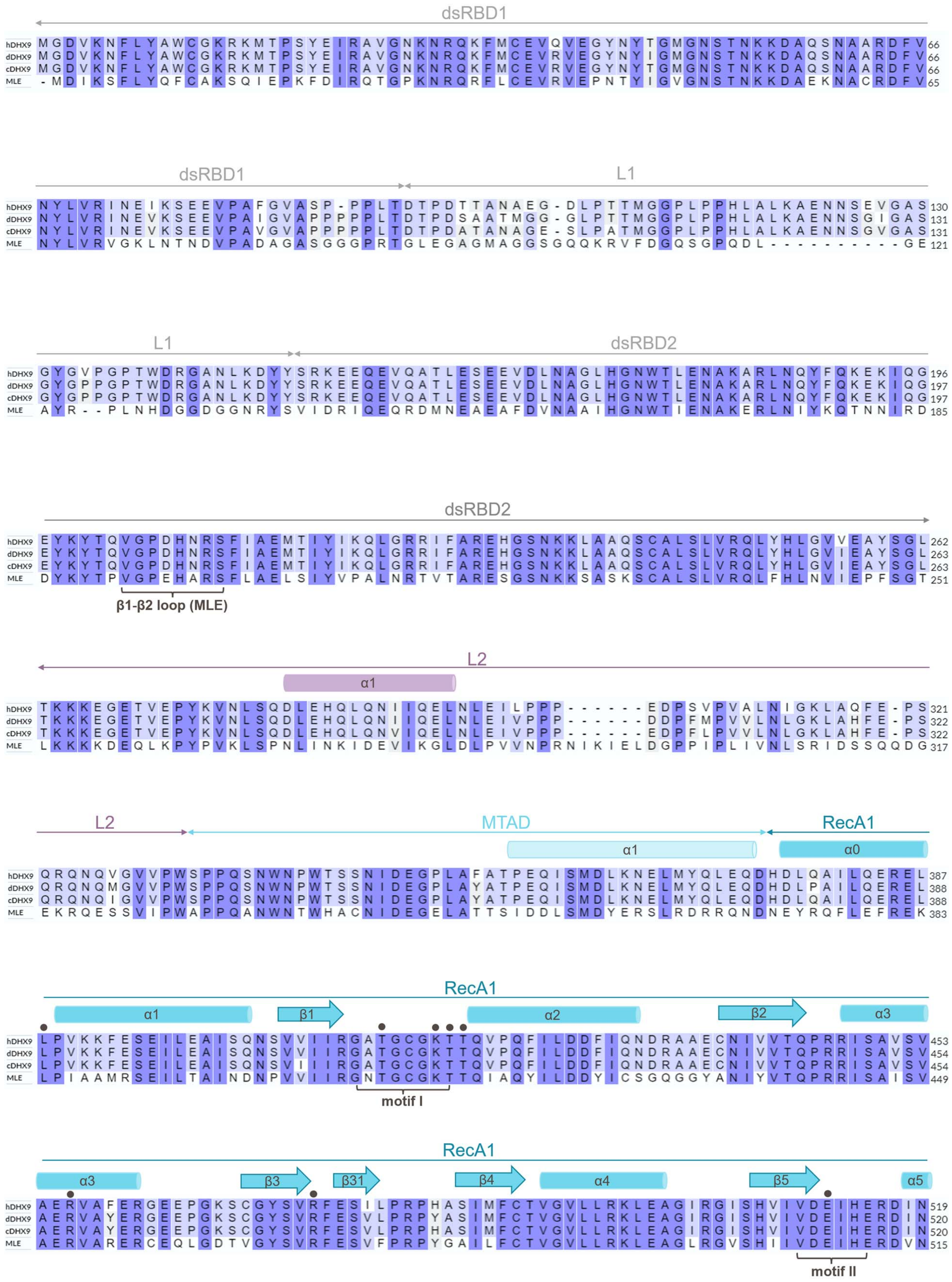
dsRBD1
hDHX9 MGDVKNFLYAWCGKRKMTPSYEIRAVGNKNRQKFMCEVQVEGYNYTGMGNSTNKKDAQSNAARDFV 66
dDHX9 MGDVKNFLYAWCGKRKMTPSYEIRAVGNKNRQKFMCEVRVEGYNYIGMGNSTNKKDAQSNAARDFV 66
cDHX9 MGDVKNFLYAWCGKRKMTPSYEIRAVGNKNRQKFMCEVRVEGYNYTGMGNSTNKKDAQSNAARDFV 66
MLE -MDIKSFLYQFCAKSQIEPKFDIRQTGPKNRQRFLCEVRVEPNTYIGVGNSTNKKDAEKNACRDFV 65
dsRBD1
L1
hDHX9 NYLVRINEIKSEEVPAFGVASP-PPLTDTPDTTANAEG-DLPTTMGGPLPPHLALKAENNSEVGAS 130
dDHX9 NYLVRINEVKSEEVPAIGVAPPPPPLTDTPDSAATMGG-GLPTTMGGPLPPHLALKAENNSGIGAS 131
cDHX9 NYLVRINEVKSEEVPAVGVAPPPPPLTDTPDATANAGE-SLPATMGGPLPPHLALKAENNSGVGAS 131
MLE NYLVRVGKLNTNDVPADAGASGGGPRTGLEGAGMAGGSGQQKRVFDGQSGPQDL----------GE 121
L1
dsRBD2
hDHX9 GYGVPGPTWDRGANLKDYYSRKEEQEVQATLESEEVDLNAGLHGNWTLENAKARLNQYFQKEKIQG 196
dDHX9 GYGPPGPTWDRGANLKDYYSRKEEQEVQATLESEEVDLNAGLHGNWTLENAKARLNQYFQKEKIQG 197
cDHX9 GYGPPGPTWDRGANLKDYYSRKEEQEVQATLESEEVDLNAGLHGNWTLENAKARLNQYFQKEKIQG 197
MLE AYR--PLNHDGGDGGNRYSVIDRIQEQRDMNEAEAFDVNAAIHGNWTIENAKERLNIYKQTNNIRD 185
dsRBD2
hDHX9 EYKYTQVGPDHNRSFIAEMTIYIKQLGRRIFAREHGSNKKLAAQSCALSLVRQLYHLGVVEAYSGL 262
dDHX9 EYKYTQVGPDHNRSFIAEMTIYIKQLGRRIFAREHGSNKKLAAQSCALSLVRQLYHLGVIEAYSGL 263
cDHX9 EYKYTQVGPDHNRSFIAEMTIYIKQLGRRIFAREHGSNKKLAAQSCALSLVRQLYHLGVIEAYSGL 263
MLE DYKYTPVGPEHARSFLAELSIYVPALNRTVTARESGSNKKSASKSCALSLVRQLFHLNVIEPFSGT 251
β1-β2 loop (MLE)
L2
α1
hDHX9 TKKKEGETVEPYKVNLSQDLEHQLQNIIQELNLEILPPP------EDPSVPVALNIGKLAQFE-PS 321
dDHX9 TKKKEGETVEPYKVNLSQDLEHQLQNIIQELNLEIVPPP------DDPFMPVVLNLGKLAHFE-PS 322
cDHX9 TKKKEGETVEPYKVNLSQDLEHQLQNVIQELNLEIVPPP------EDPFLPVVLNLGKLAHFE-PS 322
MLE LKKKKDEQLKPYPVKLSPNLINKIDEVIKGLDLPVVNPRNIKIELDGPPIPLIVNLSRIDSSQQDG 317
L2
MTAD
RecA1
α1
α0
hDHX9 QRQNQVGVVPWSPPQSNWNPWTSSNIDEGPLAFATPEQISMDLKNELMYQLEQDHDLQAILQEREL 387
dDHX9 QRQNQMGVVPWSPPQSNWNPWTSSNIDEGPLAYATPEQISMDLKNELMYQLEQDHDLPAILQEREL 388
cDHX9 QRQNQIGVVPWSPPQSNWNPWTSSNIDEGPLAYATPEQISMDLKNELMYQLEQDHDLQAILQEREL 388
MLE EKRQESSVIPWAPPQANWNTWHACNIDEGELATTSIDDLSMDYERSLRDRRQNDNEYRQFLEFREK 383
RecA1
α1
β1
α2
β2
α3
hDHX9 LPVKKFESEILEAISQNSVVIIRGATGCGKTTQVPQFILDDFIQNDRAAECNIVVTQPRRISAVSV 453
dDHX9 LPVKKFESEILEAISQNSVVIIRGATGCGKTTQVPQFILDDFIQNDRAAECNIVVTQPRRISAVSV 454
cDHX9 LPVKKFESEILEAISQNSVIIIRGATGCGKTTQVPQFILDDFIQNDRAAECNIVVTQPRRISAVSV 454
MLE LPIAAMRSEILTAINDNPVVIIRGNTGCGKTTQIAQYILDDYICSGQGGYANIYVTQPRRISAISV 449
motif I
RecA1
α3
β3
β31
β4
α4
β5
α5
hDHX9 AERVAFERGEEPGKSCGYSVRFESILPRPHASIMFCTVGVLLRKLEAGIRGISHVIVDEIHERDIN 519
dDHX9 AERVAYERGEEPGKSCGYSVRFESVLPRPYASIMFCTVGVLLRKLEAGIRGISHVIVDEIHERDIN 520
cDHX9 AERVAYERGEEPGKSCGYSVRFESVLPRPHASIMFCTVGVLLRKLEAGIRGISHVIVDEIHERDIN 520
MLE AERVARERCEQLGDTVGYSVRFESVFPRPYGAILFCTVGVLLRKLEAGLRGVSHIIVDEIHERDVN 515
motif II

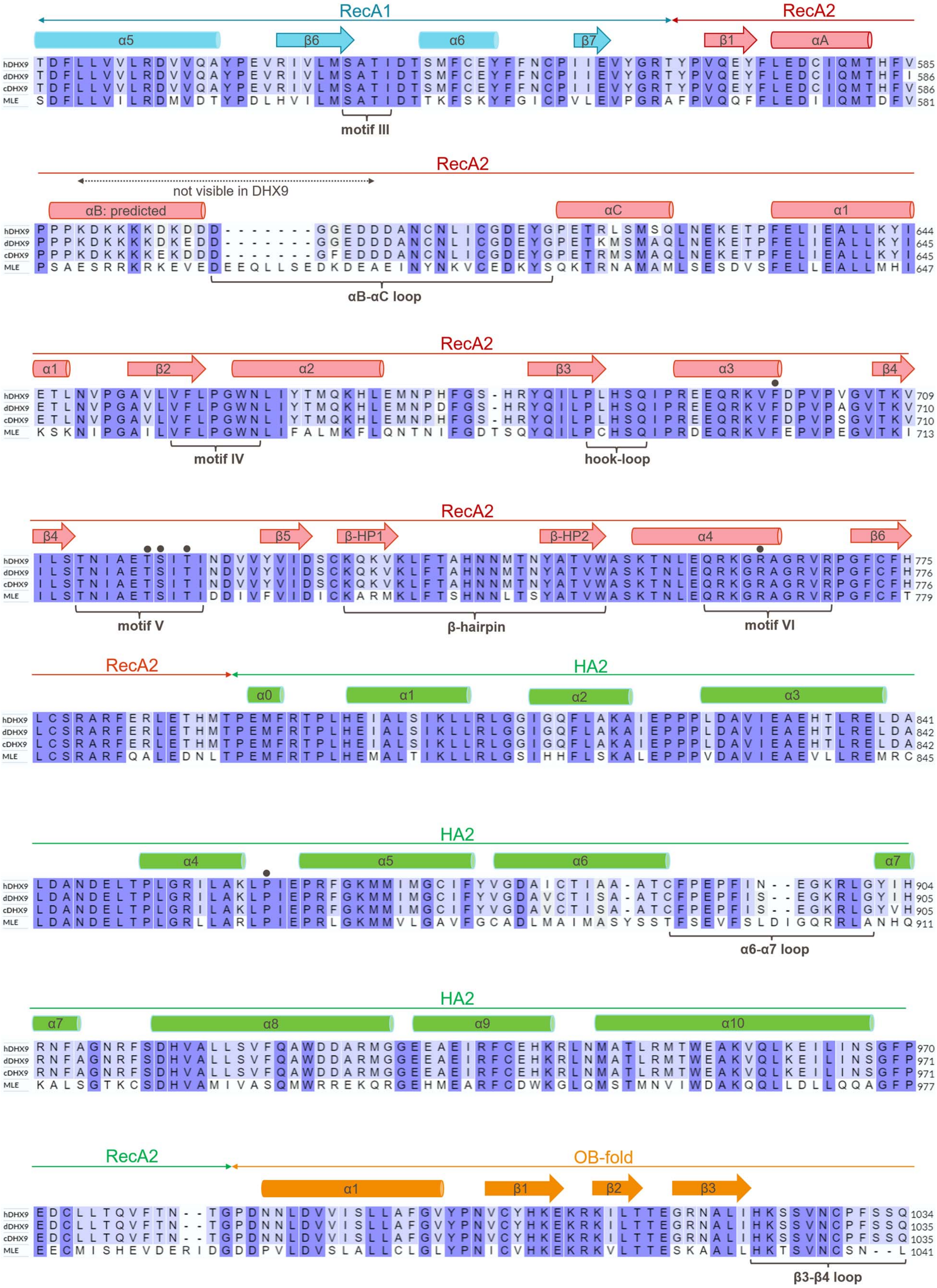
RecA1
RecA2
α5
β6
α6
β7
β1
αA
hDHX9
dDHX9
cDHX9
MLE
motif III
not visible in DHX9
αB: predicted
αC
α1
αB-αC loop
β2
α2
β3
α3
β4
motif IV
hook-loop
β5
β-HP1
β-HP2
α4
motif V
β-hairpin
motif VI
HA2
α0
α7
α6-α7 loop
α8
α9
α10
OB-fold
β3-β4 loop

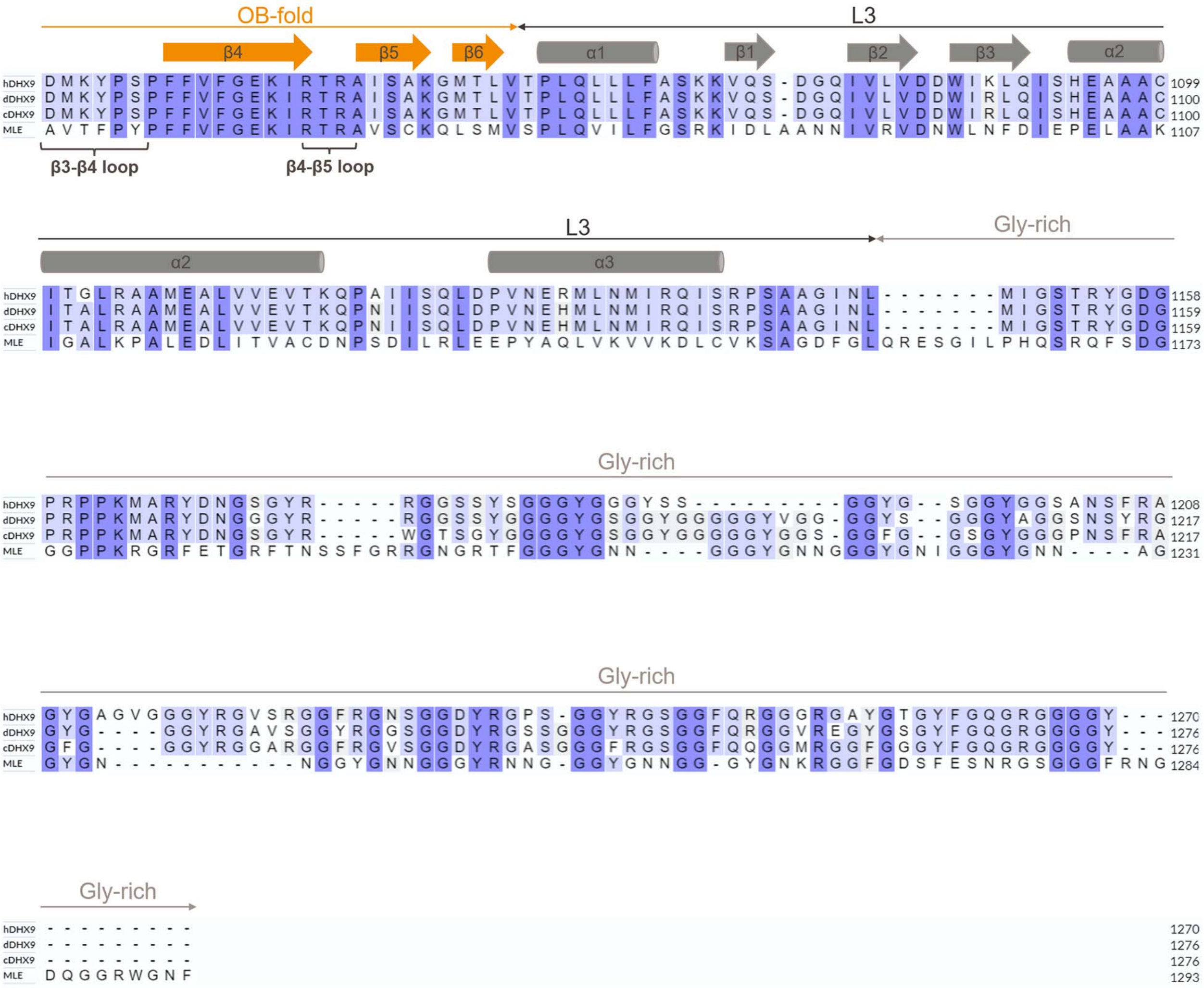

Figure S2 Sequence alignment of DHX9 sequences from human (hDHX9), dog (dDHX9), cat (cDHX9) and *Drosophila* MLE. Sequence conservation levels are indicated by color: white = low conservation, light blue = moderate conservation, blue = sequence identity. Secondary structure elements of human DHX9 (cylinder, α-helix; arrow, β-strand) are shown and mirror the domain color scheme from Figure 1. Structural features and motifs discussed in the text are indicated with brackets; Residues highlighted in the text are denoted with a black mark.

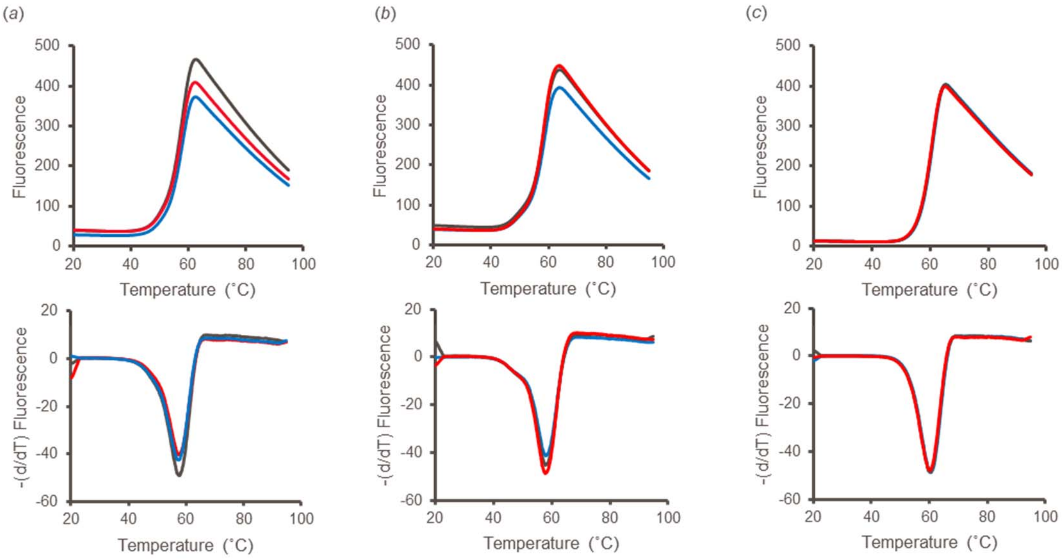

Figure S3 Melting temperature measurements of mammalian DHX9 proteins used for crystallization. Melting curves and the first derivatives plots are shown in the top and the bottom panels, respectively. (*a*) Human DHX9 (residues 150-1150); melting temperature = 57.4 ˚C. (*b*) Dog DHX9 (residues 151-1151); melting temperature = 58.7 ˚C. (*c*) Cat DHX9 (residues 151-1151); melting temperature = 60.6 ˚C.

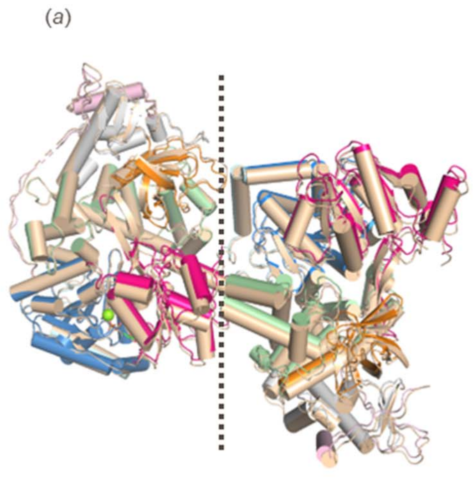

Figure S4 Packing interactions are similar between human DHX9 (space group C222$_1$) and cat DHX9 (space group P4$_3$2$_1$2). (*a*) The human DHX9 asymmetric unit (DHX9 domains colored as in Fig. 1, chain A on the left, chain B on the right) is recapitulated by cat DHX9 (wheat, left) by including one symmetry molecule (wheat, right). (*b*) Symmetry molecules for human and cat DHX9 overlap well in the extended unit cell. The same color schemes are used as in *(a)*.

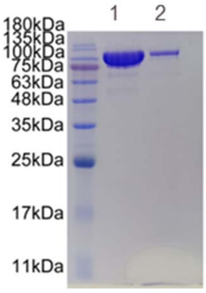

Figure S5 SDS-PAGE of human DHX9 (114.2 kDa) shows intact protein before and after crystallization. Lane 1, protein sample used for human DHX9 crystallization. Lane 2, dissolved crystals of human DHX9.

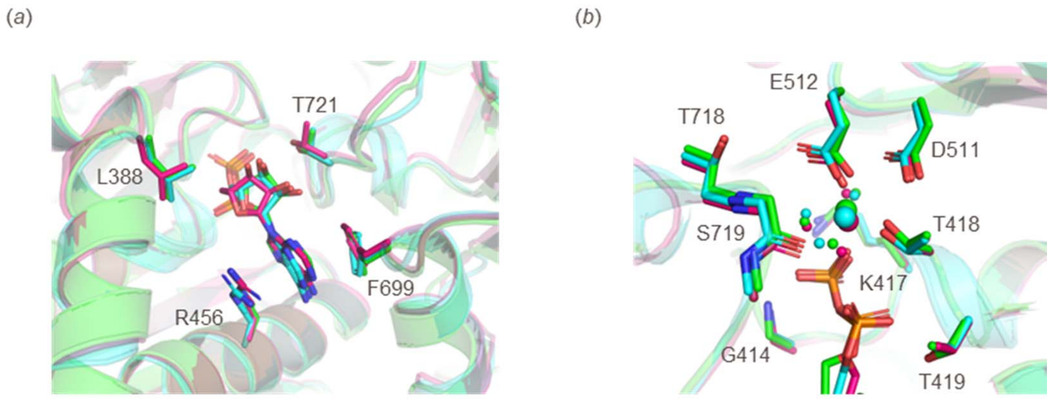

Figure S6 The nucleotide binding site is conserved across human (green), dog (cyan) and cat (magenta) DHX9. (*a*) and (*b*) correspond to the perspectives used in Figures 3d and 3e, respectively. Human residue numbers are shown.

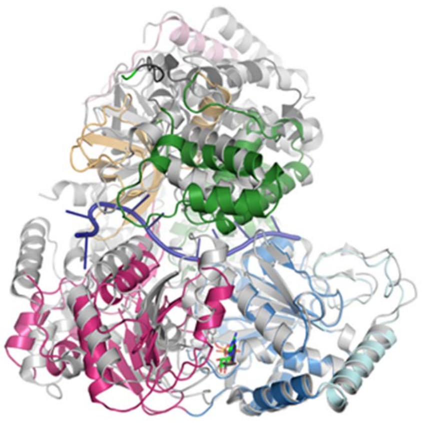

Figure S7 Superposition of DHX9 and MLE relative to the RecA1 domain. The color scheme is same as in Fig. 4a.
